# State Health Officer’s Report

**Published:** 1899-01

**Authors:** 


STATE HEALTH OFFICER'S REPORT.—Dr. W. F. Blunt, State Health Officer, has sent us a copy of his report, a neat pamphlet of ten or twelve pages. It gives an account of the operation of quarantine and a summarized report of expenses. The appropriation for 1898, $33,000, was exhausted November 30, and a deficiency of some $3,750 occurs, that sum being required to run the department to the end of the fiscal year, February 28, 1899. The State Health Officer estimates that an appropriation of $3,900 a year for the next two years will be enough to run the quarantine stations, and asks for an appropriation to construct buildings at Texarkana, Waskom and Sabine Crossing of the Southern Pacific Railroad and other points on the Louisiana border, the law requiring all persons coming to Texas from infected points during the "close season" to be detained ten days at such stations and to be fed and lodged by the State, and as there is nothing in the way of shelter at those temporary camps but tents, considerable hardship was suffered by travelers after the cold and rainy weather set in.

---

THE TEMPORARY QUARANTINE STATION at Texarkana, where several roads enter the State, necessitating the services of five inspectors and a large corps of assistants, guards, etc., cost the State $50 a day during the yellow fever scare, and the service was economically and efficiently administered by Dr. Talbot and his staff, at that.

